# Multiplexed tissue biomarker imaging

**DOI:** 10.1186/s40425-016-0115-3

**Published:** 2016-02-16

**Authors:** Edward C. Stack, Periklis G. Foukas, Peter P. Lee

**Affiliations:** Department of Life science and Technology, PerkinElmer, Hopkinton, MA USA; Center of Experimental Therapeutics and Ludwig Institute of Cancer Research, University Hospital of Lausanne, Lausanne, Switzerland; Department of Pathology, University of Athens Medical School, Attikon University Hospital, Haidari, Greece; Department of Immuno-Oncology, City of Hope, Duarte, CA USA

## Name(s) of the technology

Multiplexed Tissue Biomarker Imaging

## Description of the technology

**Fig. 1:**
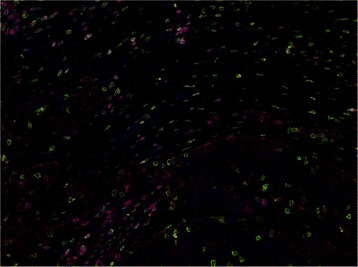
Multiplexed Lymphocyte Assay in Ovarian Adenocarcinoma. Representative TSA multiplex of CD3 (green), CD4 (red), CD8 (yellow), CD45RO (magenta), Cytokeratins (brown) and DAPI (blue) in Ovarian Cancer. Multispectral imaging yields a composite image where each marker-associated dye can be reliable separated for accurate phenotypic and expression analyses

The detection of structural and functional proteins in cells within the tumor microenvironment in tissue samples is achieved by immunolabeling with specific antibodies. These target proteins are visualized with the subsequent application of either an enzymatic reaction that induces chromogen precipitation at the site of antibody-antigen binding (immunoenzyme method) or by using fluorescent dyes (e.g., fluorophores, fluorescent quantum dot nanocrystals) conjugated either to primary or secondary antibodies (direct or indirect immunofluorescence, respectively). In order to maximize the amount of information that can be acquired from the intact tumor anatomy as well as to delineate the spatial and temporal expression information, multiplexed staining approaches are required. Serial sections lack sufficiency due to the changing tissue morphology, which prevents the accurate identification of co-expression and lacks contextual assessment [1–3]. Advanced multiplexed immunofluorescence involving iterative stain and strip procedures can offer significant multiplexing of up to 30 markers [2] and through linear alignment can report on a single region of interest. Through a more standard immunoenzyme method, the Tyramide Signal Amplification (TSA) technique allows for serial application of multiple TSA fluorophores on a single tissue section. This technique can result in multiplexes of up to 7 fluorescent dyes, which can be effectively interrogated using a multispectral microscope [3], Fig. 1.

## Type of data obtained/readout

Multiplexed tissue biomarker imaging can enable the comprehensive characterization and help to reveal the spatial relationships between stromal and immune cells within tumors. This method can label up to 6 biomarkers (plus 1 counterstain) in a single tissue section, where cells reside within a two dimensional relationship. Imaging of multiplexed samples uses morphological structures (e.g., tumor-specific cytokeratin expression) and cellular features (e.g., nuclear counterstaining) to identify all cells and their compartments (nucleus, membrane, and cytoplasm) within the tissue. The expression information for every biomarker in the multiplex is exported in table format in delimited .txt files. Subsequent analysis of the data generated by the multispectral imaging of multiplexed samples can include: phenotyping, positivity/negativity counts and H-scoring, density measurements, and spatial point pattern analyses. For example, this method allows for the assessment of FOXP3+ regulatory T cells (Tregs) in tumor tissues, which have been shown to be associated with adverse prognosis [4, 5]. In addition, multiplexed analysis of CD3, CD4, CD8, CD25, FOXP3, and Ki67 would yield a more informative assessment of Treg and cytotoxic T cell relationship in the context of CTLA-4 blockade. Moreover, the importance of the spatial distribution of immune cell infiltrates have been highlighted in a recent study, showing that the density of CD8+ T cell infiltrates in the melanoma invasive margin was the best full predictive parameter of clinical response to anti-PD-1 immunotherapy [6].

Another useful application would be the assessment of dendritic and myeloid cell types. For example, dendritic cells, known to upregulate expression of PD-L1 [7], can be subdivided into plasmacytoid and myeloid dendritic cells (pDC and mDC) [8]. In this instance, multiplexed assessment of CD123, CD11c, CD1c and CD141 may aid in the assessment of dendritic cells within the context of anti-PD1 checkpoint blockade (pDC can be defined through CD11c (low) and CD123 co-expression, while mDC populations can be characterized by CD11c and CD1c or CD11c and CD141 co-expression). Myeloid derived suppressor cells (MDSC) can also be subdivided into granulocyte and monocyte lineages, defined by CD11b, CD15, and CD14. Interestingly, as tumor MDSCs mature, they give rise to tumor-associated macrophages (TAMs) [9]. TAMs can be further subdivided into either ‘M1’ or ‘M2’ based on their differential responses to certain cytokines [10]. In practice, M1 and M2 can be profiled through CD68 and CD163. Given the potential role for TAMs in immune suppression in classic Hodgkin’s lymphoma (cHL), PD-L1 upregulation in CD68+ TAMs was recently shown to occur only when these cells were in close proximity to CD30+ PD-L1+ Reed-Sternberg cells [11]. These findings demonstrate a potential mechanism for checkpoint blockade, especially in light of the recent clinical findings of significant clinical benefit of PD-1 checkpoint blockade in advanced/refractory cHL [12].

Tumor-draining lymph nodes (TDLNs) have also been analyzed using this approach. In doing so, immune profile changes within TDLNs have been shown to correlate with clinical outcome [13–15]. Moreover, in a recent study, multispectral imaging of CD3, CD8, FOXP3, CD163, and PD-L1 was used to analyze the tumor microenvironment as a predictor of the successful generation and expansion of autologous tumor-reactive tumor infiltrating lymphocytes (TIL) in melanoma patients. Using this panel, it was shown that the CD8+:FOXP3+ ratio was a strong predictor of the successful generation of TIL. In addition, incorporating measures of CD163+ macrophages and the CD8+:PD-L1+ ratio increased the negative predictive value of this immunoprofile, which, if validated, could be used as a predictive biomarker to guide immunotherapy in melanoma patients [16].

## Limitations of the approach

Because multiplexed analyses on tissue are not common, there is risk that a lack of biological information could result from a multiplexed tissue biomarker imaging of 6 targets. Thus, the results may not correlate with clinical status or outcome. As a result, this approach could be rendered insufficient. While deeper fluorescent multiplexing has been demonstrated [2, 17], the multiplexed tissue biomarker imaging method is similar to both standard immunoenzyme assays and TSA multiplexing. Therefore, it has a more familiar protocol that could ease its adoption. More recently, enhanced multiplexing has been reported with antibodies labeled with metal isotopes and analyzed by mass cytometry-based approaches [18, 19]. However, although the morphology should be preserved with immunolabeling, there are concerns with using this approach that the preservation of the discrete microenvironment morphology may be compromised due to lack of resolution compared to that which is currently achieved with standard optical microscopy.

## Types of samples needed, special issues pertaining to samples, and sample analyses

Tissue for this assay can either be fixed in formalin or flash-frozen. Fixation artifact from formalin-fixed paraffin embedded (FFPE) tissues, such as autofluorescence, can be abrogated via autofluorescence reduction using multispectral imaging. In this method, the spectral properties of autoflourescence can be isolated from all other relevant informative spectra. Optimization of the multiplexed staining is required to avoid spectral crosstalk that could complicate target unmixing, which is typically achieved through use of similar tissue types (for many immune markers, tonsil makes an excellent control tissue). Additional care must also be put into both image analysis, as well as the downstream data analysis, where large datasets can be obtained. This would include involving bioinformatic support, where use of advanced statistical scripting capacity would further interrogation of multiparametric data, such as that obtained from spatial point pattern assessments between varied cell phenotypes.

## Level of evidence

Multiplexed tissue biomarker imaging has undergone many years of development, while recent advances have been published within the last year [3, 11, 16, 20]. Multispectral tissue imaging has over 700 publications over the last ten years. The entire application – multiplexed staining currently up to 7 fluorescent dyes and multispectral imaging – is offering and will continue to offer novel, tissue-based contextual clues in cancer immunology.
